# The reporting of uveal melanoma cases to the cancer registry in North Carolina

**DOI:** 10.3389/fonc.2022.877599

**Published:** 2022-08-03

**Authors:** Kathleen G. Gordon, Richard D. Carvajal, Sharnee’ L. Graham, Odette M. Houghton

**Affiliations:** ^1^ Immunology and Internal Medicine, IQVIA, Durham, NC, United States; ^2^ Division of Hematology/Oncology, Columbia University Irving Medical Center, New York, NY, United States; ^3^ Brody School of Medicine, East Carolina University, Greenville, NC, United States; ^4^ Department of Ophthalmology, Mayo Clinic Arizona, Phoenix, AZ, United States

**Keywords:** Uveal melanoma, cancer, registry, North Carolina, abstraction, ophthalmology

## Abstract

**Objective:**

To ascertain the completeness of reporting of uveal melanoma cases in North Carolina to the state’s cancer registry.

**Methods:**

This was a retrospective chart review performed at a single institution analyzing the completeness of information reported to the North Carolina Cancer Registry between 2010 and 2015. A list of all patients with uveal melanoma diagnosed, treated and/or followed at UNC-Chapel Hill between 2010-2015 was compared to the list of patients with uveal melanoma reported to the North Carolina Central Cancer registry during the same time frame.

**Results:**

Based on ICD 9 and 10 codes, there were 66 patients with ciliary body or choroidal melanomas diagnosed, followed and/or treated at UNC between 2010 and 2015. Of those, 41 (62%) were on the list of cases reported through the UNC Cancer Registry to the NCCCR. A chart review of the excluded cases was performed and the following barriers to reporting of uveal melanoma were identified: lack of diagnostic imaging results, lack of histopathologic confirmation, inconsistent language used to communicate diagnosis, and lack of implementation of the North American Association of Central Cancer Registries’ National Interstate Data Exchange Agreement.

**Conclusion:**

The diagnosis and treatment of uveal melanoma is unique when compared to other types of cancers. Diagnosis is based on clinical features and characteristic findings on ophthalmic imaging and ultrasound. There is often no pathology report or radiologic imaging which makes it difficult for hospital registrars to recognize and confirm cases of uveal melanoma. This creates significant barriers to reporting cases to state and national cancer registries. The incomplete data makes it difficult to detect changes in the incidence of uveal melanoma in North Carolina. The development of a national uveal melanoma registry should be seriously considered.

## Introduction

National and Regional Cancer Registries provide population-based cancer incidence data on which to base national, state, and local health planning (1, 2). These registries also serve to detect changes in the rate of cancer related death. The North Carolina Central Cancer Registry (NCCCR) is a repository of population-based cancer incidence data for the state of North Carolina. Analysis of the information collected through the registry helps to detect demographic and geographic factors affecting risk. It is also used to develop strategies for the prevention, treatment, and control of various forms of cancer. In North Carolina, facility registrars are responsible for reporting cases of cancers to the NCCCR.

Uveal melanoma is the most common primary intraocular tumor in adults occurring in approximately 5.2 patients per million per year (3). According to the data available through the Surveillance Epidemiology and End Results (SEER) Program of the National Cancer Institute, the incidence, and demographics of uveal melanoma in the United States has not changed in 41 years.

This study evaluated the completeness of reporting of uveal melanoma in North Carolina in response to a public health concern raised in 2014 after 5 young women who had lived in the same small town in North Carolina were diagnosed with uveal melanoma within a short period of time. The young women learned about each other through their medical oncologists in Philadelphia, including Dr. Marlana Orloff and through social media (4). The North Carolina Central Cancer Registry (NCCCR) was contacted because of the unexpected number of young women from the same area who had been diagnosed with a relatively rare ophthalmic cancer. However, the registry could not confirm an increase in the incidence of uveal melanoma in the county where the women resided. After further investigation, Dr. Orloff discovered that none of the young women’s names were included in the NCCCR or any other state cancer registry largely because they had been diagnosed and treated in different states, which is not uncommon for uveal melanoma. As a result, the authors conducted this retrospective study at a single institution with the help of that institution’s cancer registrars and the North Carolina State Registrars to explore the process of reporting of ocular melanoma and identify potential barriers.

## Methods

This study was performed at the University of North Carolina at Chapel Hill (UNC-CH.) An IRB approved chart review was undertaken to evaluate the completeness of reporting of uveal melanomas that were diagnosed, treated and/or followed at UNC-CH to the NCCCR. ICD9 and ICD10 codes were used to identify cases seen between 2010-2015. The ICD9 and 10 codes were 190.0, 190.5, 190.6, 190.9, C69.2, C69.3, C69.4 and C69.9. For the purposes of this study, uveal melanoma included ciliary and choroidal melanoma, not iris melanoma. The tumor registrars at UNC-CH provided a list of patients diagnosed, treated and/or followed at UNC-CH with a diagnosis of all ocular cancers that were reported to the NCCCR between these same dates, including uveal melanoma. The two lists were compared, and the differences were identified. The results were discussed with the UNC hospital cancer registrars. One of the hospital registrars who had experience abstracting ophthalmology cases reviewed the electronic medical records of the cases that were not reported to the NCCCR. Possible reasons for exclusion were provided. Unfortunately, there was no documentation to explain why these cases were not reported so quantification of the reasons they were excluded was not possible.

## Results

There were 66 patients diagnosed, followed and/or treated with ciliary body or choroidal melanomas at UNC-CH between 2010 and 2015. Of those, 41 (62%) were on the list of cases reported through the UNC-CH Cancer Registry to the NCCCR. Therefore, 25 of 66 cases of uveal melanoma cases diagnosed, followed and/or treated at UNC-CH were not reported to the NCCCR. The North Carolina State Registrars reviewed the missing cases and identified 2 of them in the state database that may have been reported through another hospital.

A retrospective chart review of the excluded cases was performed by a hospital registrar and one of the authors (KG) to identify reasons that cases might not have been reported. The reasons included lack of diagnostic imaging such as CT or MRI, absence of histopathologic confirmation and inconsistent language used to communicate diagnosis. Also, because the North American Association of Central Cancer Registries’ National Interstate Data Exchange Agreement (5) had not been implemented, the location of the patient’s diagnosis and treatment may have led to exclusion if the patient was diagnosed or treated outside of North Carolina. There was no data available explaining why each case was not reported. Quantification of the reasons for exclusion was not possible.

## Discussion

The purpose of cancer registries is to collect and maintain information about reportable cases of cancer, one of the leading causes of death in the United States. The Surveillance, Epidemiology and End Results (SEER) Program of the National Cancer Institute is an authoritative source of information on national cancer incidence (6). Case ascertainment began January 1, 1973 and its reach was expanded so that presently, cancer reporting occurs for well-defined population subgroups that together are comparable to the general population in the United States (**Table 1**). Registries are also maintained at a state level and this information is reported to the CDC’s National Program for Cancer Registries and the North American Association of Central Cancer Registries. The data from these cancer registries help to identify geographic and demographic factors associated with increased risk for the development of different types of cancer which can then help to develop strategies for prevention and treatment.

**Table 1:** Location of SEER Registries and year the Registries entered the SEER Program.

SEER 9 Registries	Entered SEER Program
**Data collected from 1973 and later**	
Connecticut	1973
Detroit	(no longer in the program)
Hawaii	1973
Iowa	1973
New Mexico	1973
San Francisco-Oakland	1973
Utah	1973
**Data Collected from 1975 and later**	
Seattle-Puget Sound	1974
Atlanta	1974
**SEER 13 Registries**	
**Data available from all cases diagnosed from 1992 and later**	
Los Angeles	1992
San Jose-Monterey	1992
Rural Georgia	1974
Alaska Native Tumor Registry	1999
**SEER 18 Registries**	
**Data available from all cases diagnosed from 2000 and later**	
Greater California	2001
Greater Georgia	2010
Kentucky	2001
Louisiana	2001
New Jersey	(no longer in the program)
**SEER 21 Registries**	
**Data available from all cases diagnosed from 2000 and later**	
New York	2018
Idaho	2018
Massachusetts	2018

According to the SEER program database, the incidence of uveal melanoma in the United States was unchanged between 1973 and 2013 (3). The incidence of primary uveal melanoma in the US is approximately 5.2 per million/year with the lowest incidence rate in Hawaii at 1.0 per million and highest in Iowa at 6.6 per million. However, except for Hawaii, when comparing the incidence rate of uveal melanoma in different states, there was no significant correlation with the latitudinal location of the registry. The incidence rate per million for Atlanta was 4.2, New Mexico 3.1, San Francisco 4.0, Utah 4.6, Detroit 3.3, Connecticut 3.5, and Seattle 5.8 (7). The SEER registry database does not include cancer cases treated in North Carolina. It is also important to note that the SEER registry does not include cases of uveal melanoma treated in Florida, Pennsylvania, and until recently, New York or Massachusetts where major ocular oncology referral centers are located (**Table 1**). Therefore, the detection of changing patterns in the demographics of uveal melanoma in North Carolina and several of its surrounding states relies on the data collected by their state registries.

The North Carolina Central Cancer Registry (NCCCR) collects data for all cancer cases diagnosed or treated in North Carolina including tumors of the eye and ocular adnexa. The data is used for research to investigate the causes of cancer and to evaluate geographic and behavioral risk in North Carolina. The NCCCR database relies on facilities that participate in the diagnosis, staging, treatment, continuing care, progression of disease or recurrence of any case meeting the North Carolina definition of cancer to report cases. The collection of accurate and complete data is reliant on hospital registrars’ rigorous chart reviews to confirm the diagnosis of cancer prior to reporting. Disease confirmation is based on information contained in the patient’s medical record including the visit notes, radiologic imaging reports and pathology reports.

The process of diagnosing uveal melanoma is different than for many other types of cancer since there is often no confirmatory pathology or radiologic imaging. Instead, diagnosis is based on clinical features and characteristic findings on ophthalmic imaging and ultrasound, making it difficult for hospital registrars to recognize and confirm these cases (8). This study revealed that nearly one third of cases of ciliary body or choroidal melanomas evaluated at UNC-CH between 2010 and 2015 were not reported to the NCCCR. Based on the chart review performed in collaboration with a UNC hospital registrar, possible reasons for exclusion were the language used to describe the tumor, absence of CT or MRI, lack of a pathology report, the place of residence during diagnosis and geographic location of treatment.

This study demonstrates the possibility that uveal melanoma cases are underrepresented in the North Carolina State Registry. One of the weaknesses of this study is that it is based on a retrospective chart review. Also, quantification of the reasons for exclusion was not possible since this information was not available. However, the chart review and discussion with the hospital registrars helped reveal some of the barriers to complete reporting of this relatively rare tumor. We are deeply grateful for the help we received from the UNC hospital registrars in this project. Future efforts to improve the system of abstracting cases of uveal melanoma and ensure complete reporting should include increased collaboration between ophthalmologists and cancer registrars. Also, there should be an increased effort to share data about tumors of the eye and ocular adnexa diagnosed and treated across state lines. On a larger scale, it is important to note that the information contained in the SEER registry data excludes several of the major ocular oncology referral centers in the US so important data about uveal melanoma incidence could be missing from this source as well. For these reasons, we believe that the development of a national uveal melanoma specific registry should be considered.

## Data availability statement

The original contributions presented in the study are included in the article. Further inquiries can be directed to the corresponding author.

## Author contributions

All authors have contributed to and have read the final copy of the manuscript.

## Conflict of interest

The authors declare that the research was conducted in the absence of any commercial or financial relationships that could be construed as a potential conflict of interest.

## Publisher’s note

All claims expressed in this article are solely those of the authors and do not necessarily represent those of their affiliated organizations, or those of the publisher, the editors and the reviewers. Any product that may be evaluated in this article, or claim that may be made by its manufacturer, is not guaranteed or endorsed by the publisher.

## References

[1] . Available at: https://schs.dph.ncdhhs.gov/units/ccr/.

[2] . Available at: https://www.cdc.gov/cancer/npcr/index.htm.

[3] Aronow ME Topham AK Singh AD. Uveal melanoma: 5-year update on incidence, treatment, and survival (SEER 1973-2013). Ocul Oncol Pathol (2018) 4(3):145–51. doi: 10.1159/000480640 PMC5939716 29765944

[4] Orloff M Brennan M Sato S Shields CL Shields JA Lally S. Unique geospatial accumulations of uveal melanoma. Am J Ophthalmol (2020) 220:102–9. doi: 10.1016/j.ajo.2020.07.012 32681908

[5] . Available at: https://www.naaccr.org/national-interstate-data-exchange-agreement/.

[6] . Available at: https://seer.cancer.gov/registries/.

[7] Singh AK Topham A. Incidence of uveal melanoma in the united states: 1973-1997. Ophthalmol (2003) 110:956–61. doi: 10.1016/S0161-6420(03)00078-2 12750097

[8] Tarlan B Kiratli H. Uveal melanoma: Current trends in diagnosis and management. Turk J Ophthalmol (2016) 46(3):123–37. doi: 10.4274/tjo.37431 PMC5076295 27800275

